# Exp-Function Method for Solving Fractional Partial Differential Equations

**DOI:** 10.1155/2013/465723

**Published:** 2013-05-28

**Authors:** Bin Zheng

**Affiliations:** School of Science, Shandong University of Technology, Zibo, Shandong 255049, China

## Abstract

We extend the Exp-function method to fractional partial differential equations in the sense of modified Riemann-Liouville derivative based on nonlinear fractional complex transformation. For illustrating the validity of this method, we apply it to the space-time fractional Fokas equation and the nonlinear fractional Sharma-Tasso-Olver (STO) equation. As a result, some new exact solutions for them are successfully established.

## 1. Introduction

Nonlinear differential equations of integer order (NLDEs) can be used to describe many nonlinear phenomena such as fluid mechanics, plasma physics, optical fibers, biology, solid state physics, chemical kinematics, and chemical physics. In the research of the theory of NLDEs, searching for more explicit exact solutions to NLDEs is one of the most fundamental and significant studies in recent years. With the help of computerized symbolic computation, much work has focused on the various extensions and applications of the known algebraic methods to construct the solutions to NLDEs. There have been a variety of powerful methods. For example, these methods include the generalized Riccati subequation method [1, 2], the Jacobi elliptic function expansion [3, 4], the *F*-expansion method [5], the Exp-function method [6–9], and the (*G*′/*G*)-expansion method [10, 11].

Fractional differential equations are generalizations of classical differential equations of integer order and have recently proved to be valuable tools to the modeling of many physical phenomena and have been the focus of many studies due to their frequent appearance in various applications in physics, biology, engineering, signal processing, systems identification, control theory, finance, and fractional dynamics. In order to obtain exact solutions for fractional differential equations, many powerful and efficient methods have been proposed so far (e.g., see [12–24]). But nobody has researched the application of the Exp-function method to fractional differential equations so far to our best knowledge.

In this paper, we will apply the Exp-function method for solving fractional partial differential equations in the sense of modified Riemann-Liouville derivative by Jumarie [25]. By a certain nonlinear fractional complex transformation *ξ* = *ξ*(*t*, *x*_1_, *x*_2_,…, *x*_*n*_), a given fractional partial differential equation expressed in independent variables *t*, *x*_1_, *x*_2_,…, *x*_*n*_ can be turned into another ordinary differential equation of integer order, whose solutions are supposed to have one of the following forms:

(1) $$\begin{array}{c} u\left(\xi \right)=\frac{\sum_{p=-m_{1}}^{m_{2}}a_{p}e^{p\xi }}{\sum_{q=-n_{1}}^{n_{2}}b_{q}e^{q\xi }}\;\text{or}\;\;u\left(\xi \right)=\frac{\sum_{p=-m_{1}}^{m_{2}}a_{p}e^{ip\xi }}{\sum_{q=-n_{1}}^{n_{2}}b_{q}e^{iq\xi }}, \end{array}$$

where *m*_1_, *m*_2_, *n*_1_, and *n*_2_ are unknown positive integers that will be determined by using the balance method. Determine the highest order nonlinear term and the linear term of highest order in the ordinary differential equation and express them in terms of (3). Then, in the resulting terms, balance the highest order Exp-function to determine *m*_2_ and *n*_2_ and balance the lowest order Exp-function to determine *m*_1_ and *n*_1_.

The Jumarie's modified Riemann-Liouville derivative of order *α* is defined by the following expression [25]:

(2) Dtαf(t) ={1Γ(1−α)ddt∫0t(t−ξ)−α(f(ξ)−f(0))dξ,0<α<1,(f(n)(t))(α−n),n≤α<n+1,  n≥1.

We list some important properties for the modified Riemann-Liouville derivative as follows [25, (3.10)–(3.13)] (see also in [22–24, 26]):

(3) $$\begin{array}{c} D_{t}^{\alpha }t^{r}=\frac{\Gamma \left(1+r\right)}{\Gamma \left(1+r-\alpha \right)}t^{r-\alpha }, \end{array}$$

(4) $$\begin{array}{c} D_{t}^{\alpha }\left(f\left(t\right)g\left(t\right)\right)=g\left(t\right)D_{t}^{\alpha }f\left(t\right)+f\left(t\right)D_{t}^{\alpha }g\left(t\right), \end{array}$$

(5) $$\begin{array}{c} D_{t}^{\alpha }f\left[g\left(t\right)\right]=f_{g}^{\prime }\left[g\left(t\right)\right]D_{t}^{\alpha }g\left(t\right)=D_{g}^{\alpha }f\left[g\left(t\right)\right]{\left(g^{\prime }(t)\right)}^{\alpha }. \end{array}$$

In the following, we will apply the Exp-function method to find exact solutions for the space-time fractional Fokas equation and the nonlinear fractional Sharma-Tasso-Olver (STO) equation.

## 2. Applications of the Exp-Function Method to Fractional Partial Differential Equations

### 2.1. Space-Time Fractional Fokas Equation

 We consider the space-time fractional Fokas equation [22]

(6) 4∂2αq∂tα∂x1α−∂4αq∂x13α∂x2α+∂4αq∂x23α∂x1α+12∂αq∂x1α∂αq∂x2α +12q∂2αq∂x1α∂x2α−6∂2αq∂y1α∂y2α=0, 0<α≤1.

In [22], the author solved (6) by a proposed fractional sub-equation method based on the fractional Riccati equation and established some exact solutions for it. Now we will apply the Exp-function method to this equation. Suppose *q*(*x*, *y*, *t*) = *U*(*ξ*), where *ξ* = (*k*_1_ *x*_1_^*α*^/Γ(1 + *α*)) + (*k*_2_ *x*_2_^*α*^/Γ(1 + *α*)) + (*l*_1_ *y*_1_^*α*^/Γ(1 + *α*)) + (*l*_2_ *y*_2_^*α*^/Γ(1 + *α*)) + (*ct* ^*α*^/Γ(1 + *α*)) + *ξ*_0_, *k*_1_, *k*_2_, *l*_1_, *l*_2_, *c*, *ξ*_0_ are all constants with *k*_1_, *k*_2_, *l*_1_, *l*_2_, *c* ≠ 0. Then by the use of (5), (6) can be turned into

(7) 4ck1U′′−k13k2U(4)+k23k1U(4)+12k1k2U′2 +12k1k2UU′′−6l1l2U′′=0.

First we suppose that the solution of (7) can be expressed by

(8) $$\begin{array}{c} U\left(\xi \right)=\frac{\sum_{p=-m_{1}}^{m_{2}}a_{p}e^{p\xi }}{\sum_{q=-n_{1}}^{n_{2}}b_{q}e^{q\xi }}. \end{array}$$

To determine *m*_2_, *n*_2_, we need to balance the linear term of the highest order with the highest order nonlinear term in (7). By simple calculation, we have

(9) $$\begin{array}{c} U^{\left(4\right)}=\frac{c_{1}e^{(15n_{2}+m_{2})x}+\cdots +c_{2}e^{-(15n_{1}+m_{1})x}}{c_{3}e^{16n_{2}x}+\cdots +c_{4}e^{-16n_{1}x}},\\ UU^{\prime \prime }=\frac{c_{5}e^{(3n_{2}+2m_{2})x}+\cdots +c_{6}e^{-(3n_{1}+2m_{1})x}}{c_{7}e^{5n_{2}x}+\cdots +c_{8}e^{-5n_{1}x}}, \end{array}$$

where *c*_*i*_, *i* = 1,2, 3,4 are coefficients determined by *a*_*m*_2__, *a*_−*m*_1__, *b*_*n*_2__, *b*_−*n*_1__. Balancing the highest order Exp-function in *U* ^(4)^ and *UU*′′, we have 15*n*_2_ + *m*_2_ = 14*n*_2_ + 2*m*_2_, which implies *m*_2_ = *n*_2_. Similarly, balancing the lowest order Exp-function in *U* ^(4)^ and *UU*′′, we have 15*n*_1_ + *m*_1_ = 14*n*_1_ + 2*m*_1_, which implies *m*_1_ = *n*_1_. For simplicity, we will proceed in two selected cases.

**Case 1**

Let *m*_1_ = *n*_1_ = 1, *m*_2_ = *n*_2_ = 1. Then

(10) $$\begin{array}{c} U\left(\xi \right)=\frac{a_{-1}e^{-\xi }+a_{0}+a_{1}e^{\xi }}{b_{-1}e^{-\xi }+b_{0}+b_{1}e^{\xi }}. \end{array}$$

Substituting (10) into (7), eliminating the denominator, and collecting all the terms with the same power of *e* ^*pξ*^ together, equating each coefficient to zero, yield a set of algebraic equations. Solving these equations with the aid of the mathematical software Maple yields the following family of values of *a*_*i*_, *b*_*i*_, *i* = −1,0, 1.

*Family  1.* Consider

(11) $$\begin{array}{c} a_{1}=-\frac{b_{1}\left(k_{1}k_{2}^{3}-k_{2}k_{1}^{3}+4ck_{1}-6l_{1}l_{2}\right)}{12k_{1}k_{2}},\\ a_{0}=-\frac{b_{0}\left(-5k_{1}k_{2}^{3}+5k_{2}k_{1}^{3}+4ck_{1}-6l_{1}l_{2}\right)}{12k_{1}k_{2}},\\ a_{-1}=-\frac{b_{0}^{2}\left(k_{1}k_{2}^{3}-k_{2}k_{1}^{3}+4ck_{1}-6l_{1}l_{2}\right)}{48b_{1}k_{1}k_{2}},\;\;b_{-1}=\frac{b_{0}^{2}}{4b_{1}}, \end{array}$$

where *b*_1_, *b*_0_ are arbitrary constants.

Substituting the result above into (10), we can obtain the following exact solutions to (7):

(12) U1(ξ) =(−(k1k23−k2k13+4ck1−6l1l2)(b02e−ξ+4b12eξ)   −4b0b1(−5k1k23+5k2k13+4ck1−6l1l2))  ×(12k1k2(b02e−ξ+4b12eξ)+48b0b1k1k2)−1,

where *ξ* = (*k*_1_ *x*_1_^*α*^/Γ(1 + *α*)) + (*k*_2_ *x*_2_^*α*^/Γ(1 + *α*)) + (*l*_1_ *y*_1_^*α*^/Γ(1 + *α*)) + (*l*_2_ *y*_2_^*α*^/Γ(1 + *α*)) + (*ct* ^*α*^/Γ(1 + *α*)) + *ξ*_0_. If we especially take *b*_0_ = 2*b*_1_, then we obtain the following hyperbolic function solitary wave solution:

(13) $$\begin{array}{c} U_{2}\left(\xi \right)=-\frac{\left(k_{1}k_{2}^{3}-k_{2}k_{1}^{3}+4ck_{1}-6l_{1}l_{2}\right)}{12k_{1}k_{2}}+\frac{\left(k_{2}^{2}-k_{1}^{2}\right)}{4}\text{sec}{\text{h}}^{2}\frac{\xi }{2}. \end{array}$$

**Case 2**

Let *m*_1_ = *n*_1_ = *m*, *m*_2_ = *n*_2_ = *m*, and for simplicity, we take

(14) $$\begin{array}{c} U\left(\xi \right)=\frac{a_{-m}e^{-m\xi }+a_{0}+a_{m}e^{m\xi }}{b_{-m}e^{-m\xi }+b_{0}+b_{m}e^{m\xi }}. \end{array}$$

Substituting (14) into (7), eliminating the denominator, and collecting all the terms with the same power of *e* ^*pnξ*^ together, equating each coefficient to zero, yield a set of algebraic equations. Solving these equations yields the following result.

*Family  2.* Consider

(15) $$\begin{array}{c} a_{m}=-\frac{b_{m}\left(m^{2}k_{1}k_{2}^{3}-m^{2}k_{2}k_{1}^{3}+4ck_{1}-6l_{1}l_{2}\right)}{12k_{1}k_{2}},\\ a_{0}=-\frac{b_{0}\left(-5m^{2}k_{1}k_{2}^{3}+5m^{2}k_{2}k_{1}^{3}+4ck_{1}-6l_{1}l_{2}\right)}{12k_{1}k_{2}},\\ a_{-m}=-\frac{b_{0}^{2}\left(m^{2}k_{1}k_{2}^{3}-m^{2}k_{2}k_{1}^{3}+4ck_{1}-6l_{1}l_{2}\right)}{48b_{m}k_{1}k_{2}},\\ b_{-m}=\frac{b_{0}^{2}}{4b_{m}}, \end{array}$$

where *b*_*m*_, *b*_0_ are arbitrary constants.

Substituting the result above into (14), we can obtain the following exact solutions to (7):

(16) U3(ξ) =(−(m2k1k23−m2k2k13+4ck1−6l1l2)(b02e−ξ+4bm2eξ)   −4b0bm(−5m2k1k23+5m2k2k13+4ck1−6l1l2))  ×(12k1k2(b02e−ξ+4bm2eξ)+48b0bmk1k2)−1,

where *ξ* = (*k*_1_ *x*_1_^*α*^/Γ(1 + *α*)) + (*k*_2_ *x*_2_^*α*^/Γ(1 + *α*)) + (*l*_1_ *y*_1_^*α*^/Γ(1 + *α*)) + (*l*_2_ *y*_2_^*α*^/Γ(1 + *α*)) + (*ct* ^*α*^/Γ(1 + *α*)) + *ξ*_0_. If we especially take *b*_0_ = 2*b*_*m*_, then we obtain the following solitary wave solution:

(17) U4(ξ)=−(m2k1k23−m2k2k13+4ck1−6l1l2)12k1k2+m2(k22−k12)4sech2(m2ξ).

Now we suppose that the solution of (7) can be expressed by

(18) $$\begin{array}{c} U\left(\xi \right)=\frac{\sum_{p=-m_{1}}^{m_{2}}a_{p}e^{ip\xi }}{\sum_{q=-n_{1}}^{n_{2}}b_{q}e^{iq\xi }}. \end{array}$$

Similar to the balancing process for (8), we have *m*_1_ = *n*_1_ and *m*_2_ = *n*_2_. Similar to the above, we only consider the following two cases.

**Case 1**

Let *m*_1_ = *n*_1_ = 1, *m*_2_ = *n*_2_ = 1. Then

(19) $$\begin{array}{c} U\left(\xi \right)=\frac{a_{-1}e^{-i\xi }+a_{0}+a_{1}e^{i\xi }}{b_{-1}e^{-i\xi }+b_{0}+b_{1}e^{i\xi }}. \end{array}$$

Substituting (19) into (7), eliminating the denominator, and collecting all the terms with the same power of *e* ^*ipξ*^ together, equating each coefficient to zero, yield a set of algebraic equations. Solving these equations yields the following.

*Family  3.* Consider

(20) $$\begin{array}{c} a_{1}=-\frac{b_{1}\left(-k_{1}k_{2}^{3}+k_{2}k_{1}^{3}+4ck_{1}-6l_{1}l_{2}\right)}{12k_{1}k_{2}},\\ a_{0}=-\frac{b_{0}\left(5k_{1}k_{2}^{3}-5k_{2}k_{1}^{3}+4ck_{1}-6l_{1}l_{2}\right)}{12k_{1}k_{2}},\\ a_{-1}=-\frac{b_{0}^{2}\left(-k_{1}k_{2}^{3}+k_{2}k_{1}^{3}+4ck_{1}-6l_{1}l_{2}\right)}{48b_{1}k_{1}k_{2}},\;\;b_{-1}=\frac{b_{0}^{2}}{4b_{1}}, \end{array}$$

where *b*_1_, *b*_0_ are arbitrary constants.

Substituting the result above into (19), we can obtain the following exact solutions to (7):

(21) U5(ξ) =(−(−k1k23+k2k13+4ck1−6l1l2)(b02e−iξ+4b12eiξ)   −4b0b1(5k1k23−5k2k13+4ck1−6l1l2))  ×(12k1k2(b02e−iξ+4b12eiξ)+48b0b1k1k2)−1,

where *ξ* = (*k*_1_ *x*_1_^*α*^/Γ(1 + *α*)) + (*k*_2_ *x*_2_^*α*^/Γ(1 + *α*)) + (*l*_1_ *y*_1_^*α*^/Γ(1 + *α*)) + (*l*_2_ *y*_2_^*α*^/Γ(1 + *α*)) + (*ct* ^*α*^/Γ(1 + *α*)) + *ξ*_0_. If we especially take *b*_0_ = 2*b*_1_, then we obtain the following trigonometric function solution:

(22) U6(ξ)=−(−k1k23+k2k13+4ck1−6l1l2)12k1k2−(k22−k12)4sec2ξ2.

**Case 2**

Let

(23) $$\begin{array}{c} U\left(\xi \right)=\frac{a_{-m}e^{-im\xi }+a_{0}+a_{m}e^{im\xi }}{b_{-m}e^{-im\xi }+b_{0}+b_{m}e^{im\xi }}. \end{array}$$

Substituting (23) into (7), eliminating the denominator, and collecting all the terms with the same power of *e* ^*ipnξ*^ together, equating each coefficient to zero, yield a set of algebraic equations. Solving these equations, we obtain another family of values of *a*_*i*_, *b*_*i*_, *i* = −*m*, 0, *m*.

*Family  4.* Consider

(24) am=−bm(−m2k1k23+m2k2k13+4ck1−6l1l2)12k1k2,a0=−b0(5m2k1k23−5m2k2k13+4ck1−6l1l2)12k1k2,a−m =−b02(−m2k1k23+m2k2k13+4ck1−6l1l2)48bmk1k2,b−m=b024bm.

Substituting the result above into (23), we can obtain the following exact solutions to (7):

(25) U7(ξ) =(−(−m2k1k23+m2k2k13+4ck1−6l1l2)(b02e−ξ+4bm2eξ)   −4b0bm(5m2k1k23−5m2k2k13+4ck1−6l1l2))  ×(12k1k2(b02e−ξ+4bm2eξ)+48b0bmk1k2)−1,

where *ξ* = (*k*_1_ *x*_1_^*α*^/Γ(1 + *α*)) + (*k*_2_ *x*_2_^*α*^/Γ(1 + *α*)) + (*l*_1_ *y*_1_^*α*^/Γ(1 + *α*)) + (*l*_2_ *y*_2_^*α*^/Γ(1 + *α*)) + (*ct* ^*α*^/Γ(1 + *α*)) + *ξ*_0_. If we especially take *b*_0_ = 2*b*_*m*_, then we obtain the following solitary wave solution:

(26) U8(ξ)=−(−m2k1k23+m2k2k13+4ck1−6l1l2)12k1k2−m2(k22−k12)4sec2(m2ξ).

**Remark 1**

Compared with the results in [22], the established solutions by (12), (16), (21), and (25) are new exact solutions to the space-time fractional Fokas equation, while the solutions by (13), (17), (22), and (26) are new solitary wave solutions.

### 2.2. Nonlinear Fractional Sharma-Tasso-Olver (STO) Equation

We consider the nonlinear fractional Sharma-Tasso-Olver (STO) equation with time-fractional derivative [26, 27]:

(27) $$\begin{array}{c} D_{t}^{\alpha }u+3au_{x}^{2}+3au^{2}u_{x}+3auu_{xx}+au_{xxx}=0,\;0<\alpha \leq 1. \end{array}$$

In [26], the author solved (27) by the first integral method and obtained some hyperbolic function and trigonometric function solutions, while in [27], the variational iteration method, the Adomian decomposition method, and the homotopy perturbation method are used for obtaining a rational approximation solution for (27). Now we will apply the Exp-function method to (27). To begin with, we suppose *u*(*x*, *t*) = *U*(*ξ*), where *ξ* = *kx* + (*ct* ^*α*^/Γ(1 + *α*)) + *ξ*_0_, *k*, *c*, *ξ*_0_ are all constants with *k*, *c* ≠ 0. Then by use of (5), (27) can be turned into

(28) $$\begin{array}{c} cU^{\prime }+3ak^{2}U^{\prime 2}+3akU^{2}U^{\prime }+3aUU^{\prime \prime }+aU^{\prime \prime \prime }=0. \end{array}$$

First we suppose that the solution of (28) can be expressed by

(29) $$\begin{array}{c} U\left(\xi \right)=\frac{\sum_{p=-m_{1}}^{m_{2}}a_{p}e^{p\xi }}{\sum_{q=-n_{1}}^{n_{2}}b_{q}e^{q\xi }}. \end{array}$$

Similar to the previous subsection, by balancing the highest order Exp-function in *U*′′′ and *U* ^2^ *U*′, we have *m*_2_ = *n*_2_, while we obtain *m*_1_ = *n*_1_ by balancing the lowest order Exp-function in *U*′′′ and *U* ^2^ *U*′. Similar to the solving process for the space-time fractional Fokas equation, we will also proceed the computation under two selected cases.

**Case 1**

Let *m*_1_ = *n*_1_ = 1, *m*_2_ = *n*_2_ = 1. Then

(30) $$\begin{array}{c} U\left(\xi \right)=\frac{a_{-1}e^{-\xi }+a_{0}+a_{1}e^{\xi }}{b_{-1}e^{-\xi }+b_{0}+b_{1}e^{\xi }}. \end{array}$$

Substituting (30) into (28), eliminating the denominator, and collecting all the terms with the same power of *e* ^*pξ*^ together, equating each coefficient to zero, yield a set of algebraic equations. Solving these equations yields two families of values for *a*_*i*_, *b*_*i*_, *i* = −1,0, 1.

*Family  1.* Consider

(31) $$\begin{array}{c} a_{1}=\frac{b_{1}\left(3ak^{2}\mp \sqrt{-3a^{2}k^{4}-3akc}\right)}{3ak},\;\;a_{0}=0,\;\;b_{0}=0,\\ a_{-1}=-\frac{b_{-1}\left(2ak^{3}\pm 2k\sqrt{-3a^{2}k^{4}-3akc}-c\right)}{3ak^{2}\pm \sqrt{-3a^{2}k^{4}-3akc}}, \end{array}$$

where *b*_1_, *b*_−1_ are arbitrary constants. 

*Family  2.* Consider

(32) $$\begin{array}{c} a_{1}=\frac{b_{1}\left(3ak^{2}\mp \sqrt{-3a^{2}k^{4}-3akc}\right)}{3ak},\\ a_{0}=\frac{b_{0}\left(3ak^{2}\mp \sqrt{-3a^{2}k^{4}-3akc}\right)}{3ak},\\ a_{-1}=\frac{b_{-1}\left(9ak^{3}\pm k\sqrt{-3a^{2}k^{4}-3akc}\;+3c\right)}{6ak^{2}\pm 3\sqrt{-3a^{2}k^{4}-3akc}}, \end{array}$$

where *b*_1_, *b*_0_, *b*_−1_ are arbitrary constants.

Substituting the results above into (30), we can obtain the following exact solutions to (28):

(33) U1(ξ)=(−b−1(2ak3±2k−3a2k4−3akc−c)3ak2±−3a2k4−3akce−ξ  +b1(3ak2∓−3a2k4−3akc)3akeξ) ×(b−1e−ξ+b1eξ)−1,

(34) U2(ξ)=(b−1(9ak3±k−3a2k4−3akc+3c)6ak2±3−3a2k4−3akce−ξ  +b0(3ak2∓−3a2k4−3akc)3ak  +b1(3ak2∓−3a2k4−3akc)3akeξ) ×(b−1e−ξ+b0+b1eξ)−1,

where *ξ* = *kx* + (*ct* ^*α*^/Γ(1 + *α*)) + *ξ*_0_. If we especially take *c* = −*ak* ^3^, *b*_1_ = *b*_−1_ or *c* = −*ak* ^3^, *b*_1_ = −*b*_−1_ in (33), then we obtain the hyperbolic function solitary wave solutions:

(35) $$\begin{array}{c} U_{3}\left(\xi \right)=k\tanh\xi ,\\ U_{4}\left(\xi \right)=k\coth\xi . \end{array}$$

**Case 2**

Let

(36) $$\begin{array}{c} U\left(\xi \right)=\frac{a_{-m}e^{-m\xi }+a_{0}+a_{m}e^{m\xi }}{b_{-m}e^{-m\xi }+b_{0}+b_{m}e^{m\xi }}. \end{array}$$

Substituting (36) into (28), eliminating the denominator, and collecting all the terms with the same power of *e* ^*pnξ*^ together, equating each coefficient to zero, yield a set of algebraic equations. Solving these equations yields the following results.

*Family  3.* Consider

(37) $$\begin{array}{c} a_{m}=\frac{b_{m}\left(3amk^{2}\mp \sqrt{-3a^{2}m^{2}k^{4}-3akc}\right)}{3ak},\;\;a_{0}=0,\;\;b_{0}=0,\\ a_{-m}=-\frac{b_{-m}\left(2am^{2}k^{3}\pm 2mk\sqrt{-3a^{2}m^{2}k^{4}-3akc}-c\right)}{3amk^{2}\pm \sqrt{-3a^{2}m^{2}k^{4}-3akc}}, \end{array}$$

where *b*_*m*_, *b*_−*m*_ are arbitrary constants. 

*Family  4.* Consider

(38) $$\begin{array}{c} a_{m}=\frac{b_{m}\left(3amk^{2}\mp \sqrt{-3a^{2}k^{4}-3akc}\right)}{3ak},\\ a_{0}=\frac{b_{0}\left(3amk^{2}\mp \sqrt{-3a^{2}m^{2}k^{4}-3akc}\right)}{3ak},\\ a_{-m}=\frac{b_{-m}\left(9am^{2}k^{3}\pm mk\sqrt{-3a^{2}m^{2}k^{4}-3akc}+3c\right)}{6amk^{2}\pm 3\sqrt{-3a^{2}m^{2}k^{4}-3akc}}, \end{array}$$

where *b*_*m*_, *b*_0_, *b*_−*m*_ are arbitrary constants.

Substituting the results above into (36), we can obtain the following exact solutions to (28):

(39) U5(ξ) =(−b−m(2am2k3±2mk−3a2m2k4−3akc−c)3amk2±−3a2m2k4−3akce−mξ   +bm(3amk2∓−3a2m2k4−3akc)3akemξ)  ×(b−me−mξ+bmemξ)−1,

(40) U6(ξ) =(b−m(9am2k3±mk−3a2m2k4−3akc+3c)6amk2±3−3a2m2k4−3akce−ξ   +bm(3amk2∓−3a2k4−3akc)3akemξ   +b0(3amk2∓−3a2m2k4−3akc)3ak)  ×(b−me−mξ+b0+bmemξ)−1,

where *ξ* = *kx* + (*ct* ^*α*^/Γ(1 + *α*)) + *ξ*_0_. If we especially take *c* = −*am* ^2^ *k* ^3^, *b*_*m*_ = *b*_−*m*_ or *c* = −*am* ^2^ *k* ^3^, *b*_*m*_ = −*b*_−*m*_ in (39), then we obtain the hyperbolic function solitary wave solutions:

(41) $$\begin{array}{c} U_{7}\left(\xi \right)=mk\tanh\xi ,\\ U_{8}\left(\xi \right)=mk\coth\xi . \end{array}$$

Now we suppose that the solution of (28) can be expressed by

(42) $$\begin{array}{c} U\left(\xi \right)=\frac{\sum_{p=-m_{1}}^{m_{2}}a_{p}e^{ip\xi }}{\sum_{q=-n_{1}}^{n_{2}}b_{q}e^{iq\xi }}. \end{array}$$

By the balancing process for (28), we have *m*_1_ = *n*_1_ and *m*_2_ = *n*_2_. Similar to the above, we will also select two cases for computation.

**Case 1**

Let *m*_1_ = *n*_1_ = 1, *m*_2_ = *n*_2_ = 1. Then

(43) $$\begin{array}{c} U\left(\xi \right)=\frac{a_{-1}e^{-i\xi }+a_{0}+a_{1}e^{i\xi }}{b_{-1}e^{-i\xi }+b_{0}+b_{1}e^{i\xi }}. \end{array}$$

Substituting (43) into (28), eliminating the denominator, and collecting all the terms with the same power of *e* ^*ipξ*^ together, equating each coefficient to zero, yield a set of algebraic equations. Solving these equations, we obtain the following two families of values.

*Family  5.* Consider

(44) $$\begin{array}{c} a_{1}=\frac{b_{1}\left(3iak^{2}\mp \sqrt{3a^{2}k^{4}-3akc}\right)}{3ak},\;\;a_{0}=0,\\ a_{-1}=-\frac{b_{-1}\left(-2ak^{3}\pm 2ik\sqrt{3a^{2}k^{4}-3akc}\;-c\right)}{3iak^{2}\pm \sqrt{3a^{2}k^{4}-3akc}}, \end{array}$$

where *b*_1_, *b*_−1_ are arbitrary constants. 

*Family  6.* Consider

(45) $$\begin{array}{c} a_{1}=\frac{b_{1}\left(3iak^{2}\mp \sqrt{3a^{2}k^{4}-3akc}\right)}{3ak},\\ a_{0}=\frac{b_{0}\left(3iak^{2}\mp \sqrt{3a^{2}k^{4}-3akc}\right)}{3ak},\\ a_{-1}=\frac{b_{-1}\left(-9ak^{3}\pm ik\sqrt{3a^{2}k^{4}-3akc}+3c\right)}{6iak^{2}\pm 3\sqrt{3a^{2}k^{4}-3akc}}, \end{array}$$

where *b*_1_, *b*_0_, *b*_−1_ are arbitrary constants.

Substituting the results above into (43), we can obtain the following exact solutions to (28):

(46) U9(ξ) =(−b−1(−2ak3±2ik3a2k4−3akc  −c)3iak2±3a2k4−3akce−iξ   +b1(3iak2∓3a2k4−3akc)3akeiξ)  ×(b−1e−iξ+b1eiξ)−1,

(47) U10(ξ) =(b−1(−9ak3±ik3a2k4−3akc  +3c)6iak2±33a2k4−3akce−iξ   +b0(3iak2∓3a2k4−3akc)3ak   +b1(3iak2∓3a2k4−3akc)3akeiξ)  ×(b−1e−iξ+b0+b1eiξ)−1

If we especially take *c* = −*ak* ^3^, *b*_1_ = *b*_−1_ or *c* = −*ak* ^3^, *b*_1_ = −*b*_−1_ in (46), then we obtain the trigonometric function solutions:

(48) $$\begin{array}{c} U_{11}\left(\xi \right)=-k\tan\xi ,\\ U_{12}\left(\xi \right)=-k\;\text{cot}\;\xi . \end{array}$$

**Case 2**

Let

(49) $$\begin{array}{c} U\left(\xi \right)=\frac{a_{-m}e^{-im\xi }+a_{0}+a_{m}e^{im\xi }}{b_{-m}e^{-im\xi }+b_{0}+b_{m}e^{im\xi }}. \end{array}$$

Substituting (49) into (28), eliminating the denominator, and collecting all the terms with the same power of *e* ^*ipmξ*^ together, equating each coefficient to zero, yield a set of algebraic equations. Solving these equations, we obtain the following two families of values.

*Family  7.* Consider

(50) $$\begin{array}{c} a_{m}=\frac{b_{m}\left(3iamk^{2}\mp \sqrt{3a^{2}m^{2}k^{4}-3akc}\right)}{3ak},\;a_{0}=0,\\ a_{-m}=-\frac{b_{-m}\left(-2am^{2}k^{3}\pm 2imk\sqrt{3a^{2}m^{2}k^{4}-3akc}-c\right)}{3iamk^{2}\pm \sqrt{3a^{2}m^{2}k^{4}-3akc}}, \end{array}$$

where *b*_*m*_, *b*_−*m*_ are arbitrary constants. 

*Family  8.* Consider

(51) $$\begin{array}{c} a_{m}=\frac{b_{m}\left(3iamk^{2}\mp \sqrt{3a^{2}k^{4}-3akc}\right)}{3ak},\\ a_{0}=\frac{b_{0}\left(3iamk^{2}\mp \sqrt{3a^{2}m^{2}k^{4}-3akc}\right)}{3ak},\\ a_{-m}=\frac{b_{-m}\left(-9am^{2}k^{3}\pm mk\sqrt{3a^{2}m^{2}k^{4}-3akc}\;+3c\right)}{6iamk^{2}\pm 3\sqrt{3a^{2}m^{2}k^{4}-3akc}}, \end{array}$$

where *b*_*m*_, *b*_0_, *b*_−*m*_ are arbitrary constants.

Substituting the results above into (49), we can obtain the following exact solutions to (28):

(52) U13(ξ) =(−b−m(−2am2k3±2imk3a2m2k4−3akc−c)3iamk2±3a2m2k4−3akce−imξ   +bm(3iamk2∓3a2m2k4−3akc)3akeimξ)  ×(b−me−imξ+bmeimξ)−1,

(53) U14(ξ) =(b−m(−9am2k3±mk3a2m2k4−3akc+3c)6iamk2±33a2m2k4−3akce−imξ   +bm(3iamk2∓3a2k4−3akc)3akeimξ   +b0(3iamk2∓3a2m2k4−3akc)3ak)  ×(b−me−imξ+b0+bmeimξ)−1.

If we especially take *c* = −*am* ^2^ *k* ^3^, *b*_*m*_ = *b*_−*m*_ or *c* = −*am* ^2^ *k* ^3^, *b*_*m*_ = −*b*_−*m*_ in (53), then we obtain the trigonometric function solutions:

(54) $$\begin{array}{c} U_{11}\left(\xi \right)=-mk\tan\xi ,\\ U_{12}\left(\xi \right)=-mk\;\text{cot}\;\xi . \end{array}$$

**Remark 2**

If we let *m* = 2, then the solutions by (41), (54) reduce to the solutions established by Lu [26, equations (55), (56), (53), (54)]. So in this way, our solutions (41), (54) are of more general forms. Moreover, we note that the solutions denoted by (33), (34), (39), (40), (46), (47), (52), and (53) are essentially different from the results in [26, 27] and are new exact solutions to the nonlinear fractional Sharma-Tasso-Olver equation so far in the literature, while the solutions (35), (48) are new solitary wave solutions to it.

## 3. Conclusions

 We have extended the Exp-function method to solve fractional partial differential equations successfully. As applications, some generalized and new exact solutions for the space-time fractional Fokas equation and the nonlinear fractional Sharma-Tasso-Olver (STO) equation have been successfully found. As one can see, this method is based on the homogeneous balancing principle. So it can also be applied to other fractional partial differential equations where the homogeneous balancing principle is satisfied.
